# Innovations using digital technologies for periodontal diagnosis and prognosis: a narrative review

**DOI:** 10.2340/aos.v85.46561

**Published:** 2026-08-11

**Authors:** Nils Benedikt Liedtke, Christian Damgaard

**Affiliations:** 1 Department of Odontology, Faculty of Health and Medical Sciences, University of Copenhagen, Copenhagen, Denmark

**Keywords:** Artificial intelligence, periodontal disease, diagnosis, prognosis, mobile health applications

## Abstract

**Objective:**

This narrative review aims to identify and evaluate the available scientific literature on digital technologies assisting clinicians in periodontal diagnosis and prognosis, including electronic dental record systems, mobile applications, consumer-engaging platforms, and image recognition technologies.

**Methods:**

A literature search was performed in PubMed/MEDLINE, supplemented by manual screening of reference lists. Literature management was supported by Covidence. Given the narrative nature of this review, studies were selected based on relevance to the review topics without application of a formal systematic screening protocol.

**Results:**

The reviewed literature demonstrates considerable progress in periodontal care supported by artificial intelligence (AI). Deep learning models, particularly convolutional neural networks, have shown diagnostic accuracy rates ranging from approximately 70–98% for periodontitis classification from dental radiographs, with some models achieving very high sensitivity for bone loss detection. Mobile health applications and gamification strategies have shown promise for improving oral hygiene behaviors and patient engagement.

**Conclusions:**

AI and digital technologies represent promising tools for periodontal care, offering the potential for enhanced diagnostic accuracy, streamlined clinical workflows, and improved patient engagement. However, significant challenges remain regarding standardization, validation in diverse populations, and integration into clinical practice. Future research should focus on conducting multicenter prospective trials, developing standardized reporting frameworks, and addressing algorithmic bias and data privacy concerns.

## GRAPHICAL ABSTRACT

**Figure:**
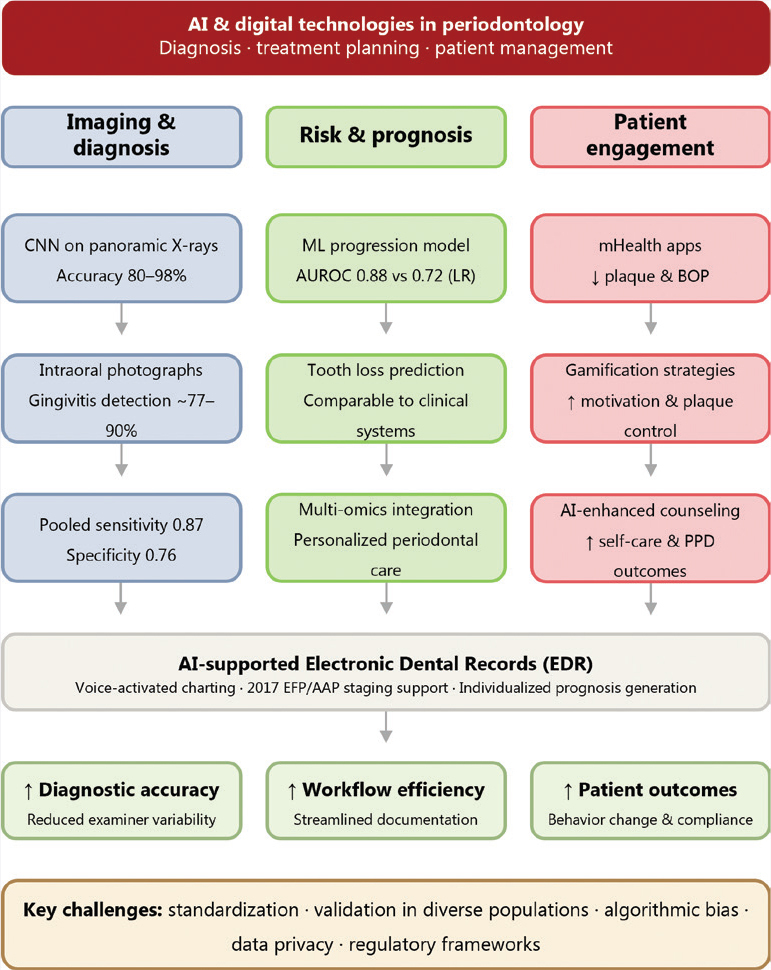

**Graphical abstract.** Overview of AI and digital technologies in periodontology The figure summarizes the three main domains covered in this review, imaging and diagnosis, risk assessment and prognosis, and patient engagement, and their integration into AI-supported electronic dental records (EDR), resulting in improved diagnostic accuracy, workflow efficiency, and patient outcomes. Key challenges to clinical implementation are highlighted at the bottom. BOP: bleeding on probing; PPD: periodontal probing depth.

## Introduction

Periodontitis is a multifactorial inflammatory disease and one of the most prevalent diseases affecting humanity, with implications for both oral and systemic health. Severe periodontitis affects approximately 1 billion people worldwide, with a global age-standardized prevalence of 12.5% (95% CI: 10.53–14.49), positioning it among the leading causes of disability globally, making it one of the most prevalent chronic inflammatory diseases worldwide [1–3]. When all severity levels are considered, prevalence rises to 33–64% in adult populations of high-income nations [4, 5].

Beyond oral health implications, periodontitis demonstrates significant associations with systemic conditions including cardiovascular disease, diabetes mellitus, respiratory diseases, and adverse pregnancy outcomes [6–8].

The landscape of periodontal diagnostics and classification underwent a paradigm shift with the 2017 World Workshop on the Classification of Periodontal and Peri-Implant Diseases and Conditions, jointly organized by the European Federation of Periodontology (EFP) and the American Academy of Periodontology (AAP) [9]. This landmark consensus replaced the previous distinction between chronic and aggressive periodontitis with a unified periodontitis category characterized by a multidimensional staging and grading system. Staging (I–IV) reflects disease severity and complexity based on clinical attachment loss, radiographic bone loss, tooth loss, and case complexity factors, while grading (A–C) provides supplemental information about disease progression rate and risk factors including smoking and diabetes [10, 11].

Artificial intelligence (AI) encompasses computational systems designed to perform tasks typically requiring human intelligence, while machine learning (ML) represents a subset enabling algorithms to learn patterns from data without explicit programming.

Deep learning (DL), particularly through convolutional neural networks (CNNs), has demonstrated remarkable capabilities in medical image analysis, pattern recognition, and predictive modeling [12, 13].

In periodontology, AI applications span multiple domains: automated radiographic analysis for bone loss quantification, periodontal charting support, clinical decision support systems, risk assessment models, treatment outcome prediction, and patient engagement platforms [14, 15]. The integration of these technologies promises to address critical challenges in periodontal care, including diagnostic variability between practitioners, time-intensive clinical assessments, and suboptimal patient compliance with oral hygiene protocols.

The primary objectives of this review are to: (1) evaluate clinical AI and digital technologies supporting periodontal assessment, periodontal diagnosis, and prognosis; (2) assess applications for electronic dental record (EDR) systems, mobile health applications, and patient-engaging platforms; (3) examine image recognition technologies for periodontal assessment; and (4) analyze AI applications for patient motivation, adherence, and behavior change.

## Methods

### Literature search strategy

This article was designed as a narrative review supported by a structured literature search. A literature search was performed in PubMed/MEDLINE to identify relevant publications on AI, ML, and DL applications in periodontology, as well as related digital health technologies including EDRs and mobile health applications. The search combined Boolean operators across two conceptual blocks: a technology block and a clinical domain block. The following search string was applied:

(‘artificial intelligence’ OR ‘machine learning’ OR ‘deep learning’ OR ‘neural network’ OR ‘convolutional neural network’ OR ‘mobile application’ OR ‘mobile health’ OR ‘mHealth’ OR ‘smartphone app’) AND (‘periodontal disease’ OR ‘periodontitis’ OR ‘periodontal diagnosis’ OR ‘periodontal risk assessment’ OR ‘alveolar bone loss’ OR ‘periodontal treatment planning’ OR ‘oral hygiene’ OR ‘gingival health’ OR ‘periodontal’)

The search was restricted to publications in English and yielded 796 records. The final search was conducted in January 2026, with eligibility limited to publications available online or formally published by 31 December 2025. Covidence was used to support reference management, deduplication, and organization of records. This review was not designed as a systematic review. Accordingly, it did not apply a formal screening protocol, independent screening by two reviewers, risk-of-bias assessment, or evidence synthesis based on the Preferred Reporting Items for Systematic Reviews and Meta-Analyses (PRISMA) guidelines. Instead, the authors selected studies thematically according to their relevance to the review objectives, prioritizing literature on AI-supported periodontal diagnosis, prognosis, EDRs, mobile health applications, and patient engagement.

Consistent with this narrative review design, no formal count of finally included studies was maintained.

Additional publications were identified through manual screening of the reference lists of included articles and relevant existing reviews.

The final selection of literature was based on relevance, methodological contribution, and thematic coverage rather than exhaustive systematic inclusion. Relevant information from selected publications was synthesized narratively, with key findings, study characteristics, and methodological details incorporated into the text as appropriate to address the review objectives.

### Eligibility criteria

#### Inclusion criteria

Studies were eligible if they: (1) investigated AI, ML, or DL applications for periodontal disease diagnosis, classification, treatment planning, or prognosis; (2) evaluated EDR systems with periodontal charting capabilities; (3) assessed mobile health applications targeting periodontal health; (4) examined consumer-engaging platforms for oral health promotion; (5) utilized image recognition technologies for periodontal assessment; (6) were published in peer-reviewed journals in English; and (7) were published between January 2010 and December 2025.

#### Exclusion criteria

Studies were excluded if they: (1) focused exclusively on non-periodontal dental conditions without periodontal components; (2) represented conference abstracts, editorials, or letters without original data; or (3) were not available in full text.

## Results

### AI for imaging data in periodontal diagnosis

#### Convolutional neural networks for bone loss detection

Radiographic assessment of alveolar bone levels remains a cornerstone of periodontal diagnosis, with panoramic and periapical radiographs serving as the most commonly employed modalities in clinical practice. The accurate measurement of the distance between the cemento-enamel junction (CEJ) and the alveolar bone crest is essential for staging periodontitis and monitoring disease progression [10, 11], yet this measurement is operator dependent and subject to considerable inter- and intra-rater variability [16]. DL methods, particularly CNNs, which are the most commonly used architectures in medical imaging, have gained considerable attention as tools for automating and standardizing the assessment of alveolar bone loss [17].

An early contribution in this area [18] applied CNNs to panoramic radiograph segments and achieved a classification accuracy of 81% for periodontal bone loss detection, notably comparable to the mean performance of six dentists at 76%. More recent work has pushed performance considerably further by applying a YOLOv8-based model (YOLOv8, ‘You Only Look Once’ version 8, a real-time deep-learning architecture that detects and localizes structures in an image in a single pass) to 2000 panoramic radiographs, achieving 97% accuracy for tooth segmentation and 94.4% overall accuracy for periodontitis staging, outperforming the periodontists’ own reported accuracy of 91.1% [19]. However, the model achieved 100% sensitivity at the expense of 0% specificity, meaning that while all diseased cases were correctly identified, healthy cases were consistently misclassified, a pattern that underscores the importance of model calibration and the continued need for professional oversight.

A further study [20] similarly demonstrated that a multitasking InceptionV3-based model (InceptionV3 and VGG-16, referenced below, are widely used CNN architectures originally developed for general image classification and commonly adapted to medical imaging tasks) could classify radiographic bone loss severity with an average accuracy of 0.87 and stable sensitivity and specificity values of approximately 0.86 and 0.88 respectively, while another investigation [21] confirmed the utility of VGG-16 (Visual Geometry Group) architectures for categorizing bone loss severity across multiple performance metrics.

Taken together, the available evidence, summarized in a systematic review and meta-analysis [22] encompassing 30 studies, points to a pooled sensitivity of 0.87 (95% CI 0.80–0.93) and specificity of 0.76 (95% CI 0.69–0.81) for AI-based periodontal bone loss detection. While these figures are encouraging, the same review noted that only 23.3% of studies achieved high-quality ratings according to APPRAISE-AI criteria, with the majority classified as intermediate quality. These findings also highlight a broader methodological point in the evaluation of dental AI systems: Strong aggregate performance measures should not be interpreted in isolation. As demonstrated by individual studies, apparently high accuracy may coincide with poor class discrimination, underscoring the need to assess clinically interpretable metrics such as sensitivity and specificity alongside overall performance. In this regard, reporting guidance for AI studies in dentistry has emphasized transparent reporting of independent test-set performance, uncertainty estimates, and metrics that are meaningful for clinical decision-making rather than reliance on accuracy alone [23]. The field is thus progressing rapidly in terms of raw diagnostic performance, but methodological rigor, including external validation, diverse training datasets, and transparent reporting, requires commensurate attention.

#### Intraoral photograph analysis

Beyond radiographic imaging, AI-based analysis of intraoral photographs represents an emerging frontier in periodontal assessment. A systematic review [24] evaluating 26 studies on this topic found wide variation in reported performance: classification studies demonstrated accuracy ranging from 0.46 to 1.00, detection studies achieved 0.56–0.78 accuracy, and segmentation approaches reported Intersection over Union (IoU) scores of 0.43–0.70. Imaging devices ranged from professional cameras and intraoral cameras to smartphones and consumer home-use devices, reflecting the breadth of clinical and non-clinical contexts being explored.

Several studies [25, 26] have shown that DL models can detect gingivitis from clinical photographs with accuracies ranging from approximately 77% to over 90%, suggesting potential for automated screening in primary care and teledentistry settings. This is a clinically meaningful prospect, given that intraoral photographs are far more accessible and less costly than radiographs. Significant challenges remain, however, particularly regarding standardization of image acquisition protocols, lighting conditions, and validation across diverse patient populations. These limitations must be addressed before such tools can be considered ready for routine clinical application.

### AI for risk assessment and prognosis

The application of ML to periodontal risk prediction and prognostication represents one of the more clinically consequential developments in the field. Rather than simply detecting disease, these approaches aim to anticipate its trajectory, enabling earlier and more targeted intervention. A longitudinal cohort study [27] illustrated this potential where a probabilistic graphic model integrating clinical data with salivary biomarkers, particularly interleukin-1 beta (IL-1β), achieved an area under the receiver operating characteristic curve (AUROC) of 0.88 for predicting periodontitis progression, significantly outperforming traditional logistic regression (AUROC 0.72). Feature importance analysis identified the number of sites with probing depth ≥ 5 mm as the strongest predictor, followed by key inflammatory biomarkers such as IL‑1β, vascular endothelial growth factor (VEGF), and matrix metalloproteinase-8 (MMP-8). These findings do not only validate the model but also reinforce existing biological understanding of disease progression.

A comparative study [28] examined ML-based prognostic models alongside established clinical periodontal prognostic systems for tooth loss prediction and found that AI-based models performed comparably overall while showing superior accuracy in specific prognostic risk categories.

A neural network model targeting the grading dimension directly assigned periodontitis grades A–C with a sensitivity of 85.7%, a specificity of 80.0%, and an overall classification accuracy of 84.2% in the training set [29]. A broader narrative review further emphasized the largely untapped potential for integrating multi-omics data (genomics, microbiome, metabolome, and proteome) to enhance predictive capabilities and enable truly personalized periodontal care [15].

### EDR systems and AI-integrated periodontal charting

The integration of AI into EDR systems represents an important area of development in periodontology, but the current evidence base differs substantially across individual components. Some technologies, such as digital periodontal charting and voice-assisted documentation, are already commercially available, whereas more advanced functions, including fully integrated risk prediction, automated treatment planning, and longitudinal patient monitoring, remain incompletely validated or largely aspirational.

**Figure 1:**
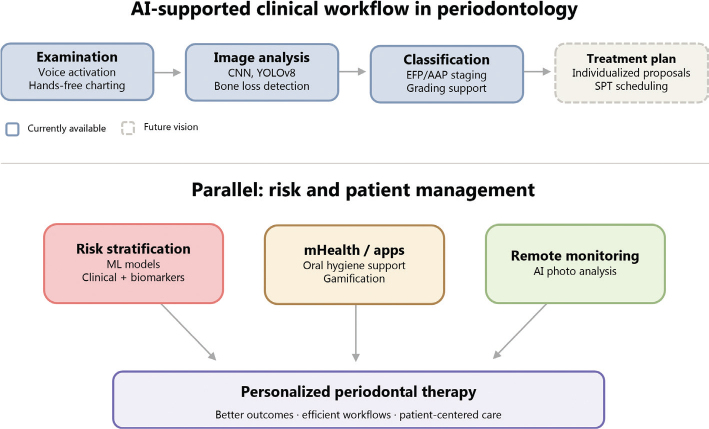
AI-supported clinical workflow in periodontology. The upper pathway shows the sequence from voice-activated examination and AI-assisted image analysis to disease classification and treatment planning. The lower pathway depicts risk stratification, mHealth support, and remote monitoring converging on personalized periodontal therapy. AI: artificial intelligence; CNN: convolutional neural networks; YOLOv8: ‘You Only Look Once’ version 8; EFP: European Federation of Periodontology; AAP: American Academy of Periodontology; SPT: supportive periodontal therapy; ML: machine learning.

At the level of clinical documentation, digital periodontal charting systems and voice-assisted tools may support hands-free recording of probing depths, bleeding on probing, recession, furcation involvement, and plaque or calculus scores [30]. These technologies should primarily be regarded as workflow-support tools that may reduce interruptions during periodontal examination and facilitate structured data entry. However, current publicly available information on specific or planned commercial systems [30, 31] is derived from company webpages or professional announcements rather than peer-reviewed clinical validation studies. Such sources are therefore cited only to illustrate current technological developments and should not be interpreted as evidence of diagnostic accuracy, prognostic performance, or clinical effectiveness. An overview of the possibilities of AI-supported clinical workflows in periodontology is provided in Figure 1.

At the diagnostic level, digital systems can support clinicians in applying the 2017 EFP/AAP staging and grading framework for periodontitis [30, 31]. The clinical rationale for such tools is clear, since staging and grading require the integration of disease severity, complexity, progression risk, and modifying factors such as smoking and diabetes [10, 11]. However, support for applying an established classification framework should be distinguished from independent validation of an AI-based diagnostic system. In other words, digital classification support may help standardize documentation, but it does not by itself demonstrate autonomous diagnostic performance.

Beyond documentation and classification, AI-based risk stratification represents a clinically relevant but still emerging field. Longitudinal modelling studies suggest that machine-learning approaches can combine periodontal parameters with additional information, including salivary biomarkers, to predict disease progression [27].

These findings support the concept of individualized risk assessment, but broader external validation across diverse clinical settings and populations is still needed before such models can be considered ready for routine integration into EDR-based decision support.

Patient-facing digital tools provide another important component of future AI-supported periodontal care. mHealth applications can deliver oral hygiene instructions, reminders, motivational feedback, and gamified elements designed to improve adherence to plaque control. Systematic review evidence suggests that mHealth interventions can reduce plaque levels and gingival inflammation [32], while evidence from a randomized controlled trial indicates that app-based support may improve bleeding on probing, oral hygiene indices, and patient knowledge or psychomotor skills [33]. However, the effects of mHealth interventions may differ across outcome domains, with clinical and behavioral improvements not necessarily accompanied by detectable short-term microbiological changes [34]. In addition, evaluations of commercially available oral-health apps show substantial variation in quality and evidence-based content [35, 36].

Remote monitoring may link patient behavior outside the clinic with clinical decision-making. In a randomized controlled trial of patients with periodontitis, at-home AI-assisted dental monitoring using smartphone-based intraoral scanning improved 3-month probing pocket depth, clinical attachment level, and plaque index compared with control care, with the strongest effects when combined with human counselling [37]. These findings support the short-term potential of AI-assisted remote monitoring although evidence remains limited regarding long-term effectiveness, scalability, image standardization, patient usability, data protection, and integration into routine EDR workflows.

### Mobile health applications for periodontal care

#### Effectiveness of mobile health interventions

Mobile health (mHealth) applications have emerged as valuable tools for periodontal patient education and self-management support.

A systematic review and meta‑analysis [32] reported significant reductions in dental plaque levels and gingival inflammation across 15 randomized trials, indicating that mHealth strategies reliably promote better oral hygiene behaviors and support gingivitis control.

Building on this broader evidence base, a randomized controlled trial [33] showed that integrating a mobile application into periodontal treatment yields substantial clinical benefits. Patients who used the app experienced notable reductions in bleeding on probing, improved plaque and oral hygiene indices, and enhanced cognitive and psychomotor skills related to oral hygiene practices.

A further publication drawing on the same clinical trial [34] but focusing specifically on microbiological outcomes found no short‑term differences between the app and control groups in subgingival microbiota diversity or composition.

**Figure 2:**
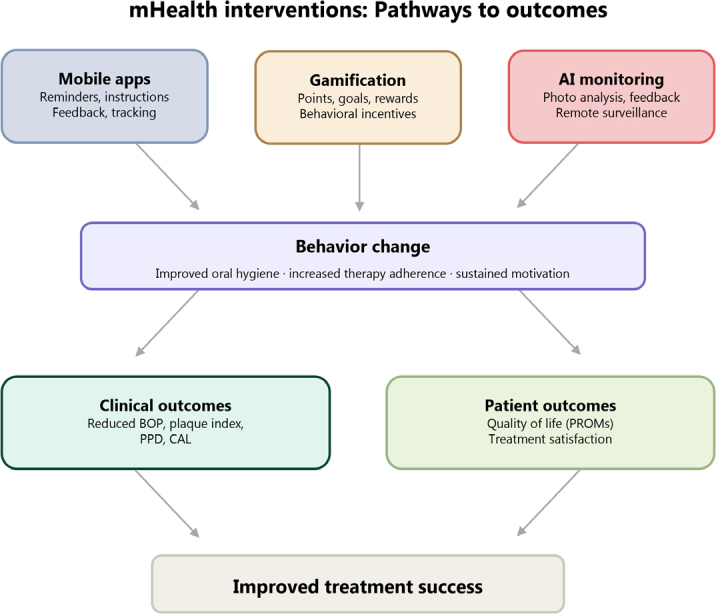
Digital patient engagement interventions and their pathways to improved treatment outcomes. Mobile apps, gamification, and AI monitoring can promote behavior change that translates into improved clinical and patient-reported outcomes, collectively contributing to periodontal treatment success. AI: artificial intelligence; BOP: bleeding on probing; PPD: periodontal probing depth; CAL: clinical attachment loss PROMs: patient-reported outcome measures.

Taken together, this evidence shows that while digitally delivered education, motivation, and reminders can strengthen oral hygiene behaviors and improve periodontal outcomes, measurable microbiological changes may require longer follow‑up periods in patients with periodontitis. The mechanisms through which these digital interventions achieve their effects and the clinical and patient-reported outcomes they target are illustrated in Figure 2.

#### Application quality and content assessment

A scoping review [38] identified 45 studies on patient‑oriented mobile oral‑health apps, with almost half focusing on improving toothbrushing behavior and 39% of randomized controlled trials reporting significant plaque reduction among app users. However, evaluations of commercially available oral‑health applications reveal substantial variability in quality. Using the Mobile App Rating Scale (MARS), one quality assessment study [39] evaluated 20 patient‑focused oral‑hygiene apps and reported a mean quality score of 3.4/5.0 (range 2.3–4.9). Their accuracy analysis showed major informational deficits: only two apps contained complete evidence‑based content across all checklist items, while most omitted key preventive guidance such as fluoride recommendations, brushing timing, rinsing practices, and interdental cleaning.

### AI and gamification in patient motivation

Gamification strategies, leveraging game design elements to enhance engagement and motivation, have demonstrated particular efficacy in oral health promotion. A comprehensive systematic review [40] evaluated 15 studies examining gamification in children’s and adolescents’ oral health care. The most prevalent gamification features included ideological incentives (83% of studies) and goal-setting mechanisms (56%). Behavior change techniques commonly incorporated included prompt intention formation (42%), providing instructions (42%), and information on behavior-health links (38%). A study examining the Brush DJ app [41] demonstrated that it significantly improved users’ perception of tooth cleanliness and brushing duration, with 88% of participants reporting increased motivation. A randomized controlled trial [42] compared a basic oral healthcare app with a gamified version, finding that while both improved mothers’ knowledge regarding children’s oral health, the gamified application produced greater reductions in children’s plaque indices, indicating superior clinical translation of behavioral change.

A recent randomized controlled trial [43] evaluated AI-enhanced health counseling for periodontitis patients. Participants were randomized to AI monitoring, AI-assisted health counseling (AIHC), or control groups. The AI-assisted groups demonstrated significantly improved self-care behaviors, plaque control records, probing pocket depths, and clinical attachment levels compared to controls. Protection motivation constructs (threat and coping appraisal) improved similarly across groups, suggesting that AI monitoring enhances behavioral translation of motivation into sustained oral hygiene practices.

The DentalMonitoring platform, utilized in several studies, enables remote patient monitoring through AI analysis of smartphone-captured intraoral photographs. The system achieved satisfactory interrater agreement with periodontists (Cohen κ = 0.8), providing real-time feedback on oral hygiene status and gingival health. This technology represents a significant advancement in extending clinical oversight beyond traditional appointment-based care models.

### Patient-related outcomes

Periodontal disease management depends not only on clinical parameters but on how patients understand and experience their condition. Since traditional measures such as probing depth are largely abstract to patients, patient-reported outcome measures (PROMs), covering quality of life, symptom burden, and treatment satisfaction, are increasingly recognized as essential complements to clinical endpoints [44].

A study on AI-generated visualizations [45] found that AI-annotated radiographs improved patients’ understanding of their condition and supported more confident clinical communication, a combination that facilitates the shared decision-making central to EFP guidelines.

Interactive digital tools are extending this patient-centered approach beyond the clinic. Based on patient-recorded photographs, the iGAM platform allows patients to receive remote feedback, which resulted in improved periodontal health over 8 weeks, with self-monitoring likely reinforcing engagement and awareness [46]. In the postoperative setting, the ExoDont app showed that structured digital follow-up significantly improves medication adherence and compliance with postoperative instructions compared to standard care [47, 48], suggesting that patients feel better supported, when actively contacted during recovery.

Most studies in this area, however, measure behavioral and clinical endpoints rather than the patient experience itself. Validated PROMs remain largely absent, a meaningful gap that future research will need to address.

## Discussion

### Principal findings

This narrative review shows that AI and digital technologies are increasingly changing how periodontal disease is diagnosed, planned, and managed. The strongest evidence comes from DL models applied to dental radiographs, where CNNs have reached diagnostic accuracy for bone loss detection that is comparable to, and in some cases exceeds, that of experienced clinicians, with advanced models achieving accuracy rates of 80–98% for tasks such as bone loss detection and CEJ identification [18, 19]. These findings carry real clinical relevance: AI-assisted diagnosis could reduce the variability between examiners, improve detection of early disease, and make clinical workflows more efficient.

Beyond imaging, the reviewed literature suggests a clear direction of travel: AI is moving from isolated tools toward integrated clinical support across risk prediction, patient motivation, remote monitoring, and documentation. The overall picture is not only of real and growing potential but also of a persistent gap between promising results in research settings and the robust, generalizable evidence that clinical practice requires.

### Imaging and diagnosis

The performance of CNN-based models for radiographic bone loss detection is encouraging. With a pooled sensitivity of 0.87 and a specificity of 0.76 across 30 studies [22], these models place AI-assisted radiographic assessment within a clinically meaningful range. At the same time, the finding that only 23.3% of these studies achieved high-quality ratings suggests that methodological rigor has not yet kept pace with the rapid development of model performance. Common limitations include retrospective single-center designs, homogeneous training datasets, and inconsistent reference standards, which together limit the generalizability of reported findings. The study using a YOLOv8-based model for panoramic radiographs assessment [19], where 100% sensitivity was accompanied by 0% specificity, provides a further reminder that a strong overall accuracy figure need not reflect balanced performance across diseased and healthy cases.

Because fewer than one in four studies met high-quality criteria, the pooled performance estimates and the conclusions drawn from them should be regarded as provisional: the aggregate figures are best read as an indication of the field’s potential rather than as confirmation of clinically validated accuracy.

AI-based analysis of intraoral photographs represents an earlier stage of development, with considerable variability in reported performance. The wide range of accuracy (0.46–1.00) [24] reflects both genuine differences in model quality and substantial heterogeneity in image acquisition protocols, outcome definitions, and study design.

The wide range of reported results suggests that performance still depends substantially on differences in image acquisition and study design, rather than reflecting a fully stable and reproducible capability. As imaging protocols become more standardized and external validation more common, this variability is likely to decrease.

But the potential of smartphone-based gingivitis detection for teledentistry and primary care screening is clinically meaningful and worth further investigation even if there are multiple acquisition protocols, which might also change over time due to the development of new image capture technologies.

### Periodontitis classification, risk assessment, and prognosis

A relevant question when evaluating AI systems for periodontal risk assessment is how well they align with the 2017 EFP/AAP classification framework. Several studies have implemented staging based on radiographic bone loss, clinical attachment loss, and complexity factors consistent with the guidelines [19, 20], which is encouraging. However, most models focus predominantly on radiographic parameters without integrating the full multidimensional staging and grading criteria, particularly disease progression rates (Grade A–C) and systemic risk factors such as smoking and glycemic control. This is arguably the single most underexploited opportunity in the field, since grading is precisely the dimension of the classification that demands the kind of multivariate pattern recognition that ML is well suited to provide.

Building on this, the application of ML to periodontal risk assessment and prognosis more broadly is an emerging and clinically promising direction, particularly given that individualized risk stratification remains challenging in everyday clinical practice. One probabilistic model [27] integrating clinical parameters with salivary biomarkers achieved an AUROC of 0.88 for predicting disease progression, outperforming logistic regression in this cohort. A further study [28] found that ML-based prognostic models performed comparably to established clinical systems for tooth loss prediction, with some advantages in specific risk categories. While these results are encouraging, they are based on individual studies with specific patient populations and clinical contexts, and broader validation will be needed before firm conclusions can be drawn. Nevertheless, the incorporation of multi-dimensional data, including longitudinal clinical parameters, biomarkers, and systemic risk factors appears to be a productive direction for future research toward more personalized periodontal care [15].

### Patient engagement

The evidence for mHealth applications and gamification in periodontal patient motivation is encouraging, though it should be interpreted with some caution. The meta-analytic findings [32] and the RCT evidence [33, 43] collectively suggest that digitally delivered education, reminders, and AI-assisted feedback can meaningfully support oral hygiene behavior in the context of periodontal treatment. Whether these short-term improvements translate into durable behavioral and clinical change over longer follow-up periods remains an open question that future research will need to address.

The quality of commercially available oral health apps is a further consideration. One app-quality assessment [39] found that only two of 20 assessed apps contained complete evidence-based content, a finding that highlights a broader pattern observable across several domains of this review: the pace of market development frequently outstrips the evidence base. As long as the regulatory framework for mHealth tools remains less stringent than for diagnostic medical devices, clinicians should exercise judgment when recommending specific applications to patients.

### Patient-reported outcomes

Although diagnostic and behavioral applications dominate the current literature, the patient’s own experience of periodontal care remains comparatively underexplored. At the same time, there is growing recognition that conventional clinical endpoints, such as probing depth, are largely abstract to patients, underscoring the importance of incorporating PROMs that capture quality of life, symptom burden, and treatment satisfaction [44]. The evidence reviewed here suggests that digital tools may contribute on this dimension: AI-annotated radiographs have been reported to improve patients’ understanding of their condition and to support more confident clinical communication [45], while interactive platforms providing remote feedback or structured postoperative follow-up have been associated with improved engagement, adherence, and self-reported support [46]. These findings are encouraging but remain preliminary, as most studies in this area measure behavioral or clinical endpoints rather than the patient experience itself. Validated PROMs are still largely absent from periodontal AI research, and their systematic incorporation represents an important direction for future work, particularly given the emphasis on shared decision-making in current EFP guidance [49].

### Implementation considerations and barriers

AI tools for periodontal care are developing rapidly in research settings, but their translation into everyday clinical practice faces substantial and partly structural obstacles.

A fundamental challenge is regulatory. The classification of AI tools depends heavily on their declared intended use, and navigating these frameworks is resource intensive, particularly for smaller developers. Additional AI-specific legislation is introducing further requirements around transparency and post-market surveillance, and the interplay between different regulatory frameworks is still being clarified. This uncertainty may partly explain why many of the promising tools described in this review remain confined to research contexts or early-adopter settings.

Data protection requirements add further complexity and connect directly to a methodological limitation already noted in Section 4.2: most published periodontal AI models have been trained on data from a limited number of centers with limited demographic diversity. Strict rules around patient consent and data sharing make it difficult to build large, representative datasets that high-performing and generalizable models require. The finding that only 23.3% of radiographic AI studies achieved high-quality ratings [22] reflects not only methodological shortcomings but also the structural constraints under which these models are developed, including external validation and questions about equitable performance across different patient populations will remain [50].

As the tools described in this review move closer to routine clinical use, building foundational AI competence into dental education will be essential for their responsible adoption.

## Limitations

This narrative review has several limitations that should be acknowledged. The restriction of the literature search to PubMed/MEDLINE, without systematic coverage of additional databases, means that relevant publications may have been missed. Although the search was structured and supported by Covidence, this was a narrative rather than systematic review, and the selection of included literature reflected the authors’ judgment and was not exhaustive. The findings should therefore be read as a critically informed overview rather than a definitive evidence synthesis. The rapidly evolving nature of the field means that commercially available products and regulatory approvals may have changed since the search was conducted. Finally, commercial information was drawn from publicly available company and regulatory sources, which may not provide independent verification of performance claims.

It remains uncertain whether these models perform reliably across different patient populations. Across both image-based diagnostic models and the clinical risk and prognostic models discussed above, most have been developed and validated on single-center datasets with limited demographic variability, and systematic reviews of DL for periodontitis note a pronounced concentration of training data within a small number of settings and patient groups [51]. The limited evidence that does speak to cross-population behavior is not reassuring. Where cross-center testing has been performed, models trained at one institution have shown markedly reduced performance when applied to data from another. In a related dental imaging task, a two-center study detecting apical lesions on panoramic radiographs reported an F1-score of 54% at the development center but only 33% at a second center in a different country, with patients’ dental status rather than image characteristics driving the drop [52]. Although this example concerns a different diagnostic target, the same dependence on center-specific data composition applies directly to periodontal models.

Such failures of generalization across populations are compounded by a second, distinct problem: even within a single dataset, an aggregate accuracy figure can mask clinically critical weaknesses. In one study, a model combined a strong overall accuracy with perfect sensitivity but zero specificity, correctly flagging every diseased case while misclassifying every healthy one [19]. Metrics derived from internal validation alone may therefore overstate both generalizability across populations and reliability within them, with the risk falling hardest on groups poorly represented in the development data.

This issue carries particular weight in periodontology because the disease itself is unevenly distributed. Periodontitis prevalence, severity, and clinical presentation differ substantially across populations, shaped by genetic susceptibility, smoking, systemic conditions such as diabetes, and access to care; prevalence and severity follow a consistent socioeconomic gradient, with higher disease burden among individuals of lower educational and income status across diverse populations [53], and at the global level, the burden falls disproportionately on low- and middle-income regions, where it continues to rise [54]. A model that encodes the characteristics of one population may consequently misclassify disease stage or miscalibrate risk when applied elsewhere. Because risk and prognostic tools directly inform treatment intensity and recall intervals, such errors have tangible clinical consequences [55]. The populations most likely to benefit from scalable, AI-assisted screening, including vulnerable groups who already face substantial barriers to dental care [56], are also those least represented in current training data, raising the prospect that these tools could widen rather than narrow existing disparities in care [54, 55].

Addressing this gap will require deliberate changes in how future studies are designed and reported. Priorities include the use of demographically and geographically diverse multicenter cohorts; routine external validation on populations distinct from the development data; and disaggregated reporting of performance across relevant subgroups rather than pooled metrics alone, so that uneven performance becomes visible rather than hidden [55]. Collaborative dataset development with institutions in underrepresented regions would further improve both the diversity of available training data and the credibility of external validation. Existing consensus-based reporting standards for AI studies in dentistry already provide a practical framework that periodontal research could adopt to make bias and equity considerations explicit [23, 55].

## Conclusion

This narrative review demonstrates that AI and digital technologies represent promising and rapidly advancing set of tools for periodontal diagnosis, treatment, and patient management. DL models, particularly CNNs, achieve diagnostic accuracy comparable to or exceeding experienced clinicians for radiographic bone loss detection and periodontitis classification.

Mobile health applications and gamification strategies show promise for enhancing patient engagement and improving clinical outcomes, though evidence for sustained behavior change requires strengthening. AI-integrated EDRs and voice-activated charting systems offer substantial efficiency gains for clinical documentation while maintaining or improving data quality.

Critical gaps remain in the evidence base, including the need for prospective multicenter validation studies, standardized reporting frameworks, assessment of cost-effectiveness, and evaluation in diverse populations. A particularly underexploited priority is the grading dimension (A–C) of the 2017 EFP/AAP classification: most current models address staging while largely neglecting grading, even though the assessment of progression rate and modifying risk factors is precisely the kind of multidimensional pattern recognition to which ML is well suited. Future research should therefore prioritize comprehensive integration of the 2017 EFP/AAP staging and especially grading criteria, development of explainable AI approaches, and longitudinal assessment of patient outcomes.

As AI technologies continue advancing, their thoughtful integration into periodontal practice holds significant potential for improving diagnostic accuracy, enhancing treatment, and ultimately improving patient outcomes. Success will require collaborative efforts among researchers, clinicians, technology developers, and regulatory bodies to ensure that these powerful tools are implemented safely, equitably, and effectively.
